# The many-to-many problem of endophenotypes in psychiatry - a biological perspective

**DOI:** 10.1038/s41380-026-03473-y

**Published:** 2026-02-09

**Authors:** Juergen Dukart, Leon D. Lotter, Casey Paquola, Simon B. Eickhoff, Leonhard Schilbach

**Affiliations:** 1 Institute of Neurosciences and Medicine, Brain & Behaviour (INM-7), Research Centre Juelich, Juelich, Germany; 2 Institute of Systems Neuroscience, Medical Faculty, Heinrich Heine University, Düsseldorf, Germany; 3 Max Planck School of Cognition, Leipzig, Germany; 4 Department of General Psychiatry 2, LVR-Klinikum Duesseldorf, Bergische Landstraße 2, 40629 Duesseldorf, Germany; 5 Department of Psychiatry and Psychotherapy, University Hospital, Ludwig Maximilians University Munich, Munich, Germany

**Keywords:** Biomarkers, Neuroscience, Psychiatric disorders

## Abstract

While modern diagnostic classification systems aim to nosologically structure psychiatric disorders, they poorly align with the genetic, neurobiological, and environmental heterogeneity observed in these disorders. This limitation has complicated the search for clinically useful biomarkers for diagnosis and treatment. Recent work on genetic and environmental contributions to mental health indicates that this heterogeneity stems from differential involvement of diverse biological pathways within and across diagnostic clusters. This complex interplay presents a many-to-many mapping problem in psychiatry, where distinct pathophysiological processes can lead to similar clinical symptoms. Here, we argue that disentangling these biological mechanisms requires development of process-specific biomarkers that could replace non-specific neuroimaging markers widely used in neuropsychiatric research. We further propose a framework for biomarker research that adopts a biologically informed perspective integrating the interactions between genes and the environment to address this problem. Such a multidimensional framework holds promise for developing biology-driven models of psychiatric disorders, enabling treatment strategies tailored to individual pathophysiology.

## Introduction

About two decades ago, Andreas Meyer-Lindenberg and Daniel Weinberger published their seminal work on the potential of neuroimaging measures as endophenotypes across psychiatric disorders [1]. These endophenotypes are supposed to represent identifiable brain circuits whose structural or functional properties are modified by risk genes associated with respective psychiatric conditions. Pathophysiological expressions of the endophenotype would then relate to observable clinical symptoms and could be used as a diagnostic or treatment response biomarker. Despite extensive research efforts in this direction, there are still no clinically established biomarkers for any psychiatric disorder. Whilst group-level neuroimaging and other biomarker alterations are repeatedly reported, the effect sizes tend to be small and of limited generalizability across different cohorts. Both limit their usability for clinical applications. Here we first discuss the current diagnostic and classification concepts in psychiatry and outline their limitations. We propose a novel framework for defining a multidimensional vulnerability search grid, mapping symptom-related biological pathways shaped by individual genetic and environmental factors. This grid can be interrogated using pathway-specific biomarkers to capture the manifestation of individual pathophysiology, offering a route toward personalized interventions.

## Diagnostic categories, symptom dimensions and their limitations

The standard diagnostic approaches in psychiatry, including DSM-5 and ICD-10 classifications, rely on the assignment of patients to distinct diagnostic categories based on their observed constellations of symptoms [2, 3]. Following this logic, two patients with limited overlap in their symptom profiles may be placed into the same diagnostic category, because they each present with a specific number of symptoms out of a longer list. Whilst this diagnostic categorization approaches for mental disorders are biologically agnostic, then agnosticism is arguably primarily a reflection of the lack of validated biomarkers that would allow for a reliable biology driven classification. This argument is underscored by the recurrent reclassification of disorders - such as anti-NMDA receptor encephalitis and Wilson’s disease - from psychiatry to neurology following the identification of reliable biomarkers. [4, 5].

In light of this, concerns have been repeatedly raised regarding the validity of many of the psychiatric diagnoses [6]. Lacking objective biomarkers not only for diagnosis, but also for treatment selection, treatment optimization is often based on trial and error with a substantial proportion of patients failing to respond to available treatments [7]. This heterogeneity of symptom constellations and treatment responses is difficult to address in the current diagnostic setting [8]. Different efforts, such as the Hierarchical Taxonomy of Psychopathology (HiTOP) approach, have aimed to advance the classification of psychopathology and maximize its usefulness for research and clinical practice by revising the current diagnostic framework into empirically derived syndromes [9]. Yet, such approaches suffer under the same assumptions as older symptom-based categorizations, in that (i) such clinical categories exist and (ii) the manifestation of a similar symptom occurs due to convergent pathophysiological mechanisms which may be caused by the same or distinct etiologies. The first assumption is essential for the meaningful development of diagnostic biomarkers, while the second is critical for effective interventions. If the first assumption does not hold, identifying a common biomarker for a specific diagnosis becomes futile. Likewise, a standardized treatment is unlikely to be effective for two patients exhibiting the same symptom, but driven by non-convergent pathophysiological mechanisms. Later, we discuss why both assumptions are unlikely to hold from genetic and environmental perspectives.

An alternative dimension-based approach is adopted by the so-called Research Domain Criteria (RDoC). RDoC aims to study mental disorders based on their underlying neurobiological mechanisms by integrating genetics, neuroscience and behavioral science [10]. The focus is hereby on understanding the neural circuitry underlying specific cognitive and symptom domains to identify biomarkers and viable treatment targets. Beside the explicit assumptions, it assumes implicitly that brain-behavior relationships are tractable and mappable (i.e. at region or circuit level) to specific clinical constructs such as acute fear or social communication. A dysfunction of such a construct is then responsible for manifestation of a specific clinical symptom dimension, irrespective of the actual diagnostic category.

The categorical approaches carry the advantage of parsing the psychiatric population into distinct clinically manageable subpopulations allowing for treatment optimization along these limited number of categories. The dimensional approaches aim for dissection of the observed symptom space into a limited number of symptom dimensions that could be more easily linked to neurobiological mechanisms, thereby allowing for targeted optimization of the treatment along these dimensions. Despite these advantages, both approaches are limited by the validity of their underlying assumptions as both do not account for the possibility of the many-to-many mapping problem in psychiatric disorders. More specifically, due to the highly multidimensional interactions of cellular and molecular mechanisms in the polyneurotransmitter landscape of the brain, the same symptom or even the same cluster of symptoms could be subserved by distinct mechanisms. Major evidence supporting this notion comes from lesion mapping studies often demonstrating limited to no overlap between lesions inducing similar neurological or psychiatric symptoms [11, 12].

This issue is further complicated by the use of rather unspecific biomarkers in most clinical studies in psychiatry. Take for example, the notion of an excitation inhibition (E/I) imbalance, which is frequently discussed in the context of autism and other psychiatric and neurological disorders [13]. In the most basic form, a presumed E/I imbalance can be achieved through direct excitation or inhibition of the GABAergic or glutamatergic neurotransmitter systems, not to mention the effect of different receptor subtypes and distinct short- and long-range projection mechanisms. Indirect modulation or compensatory shifts in E/I balance are also possible through or in response to known interactions with other neurotransmitter systems [14]. Moreover, functional E/I measures to date are largely based on modelling of E/I imbalance from unspecific imaging modalities such as resting state functional magnetic resonance imaging or electroencephalography. Both do not differentiate between different neurotransmitter systems nor between excitatory, inhibitory or modulatory signals. Modelling of the E/I ratio based on such measures is therefore inherently limited by the underlying assumptions reducing the plethora of possible mechanisms to a single number. Such a manifold of possible mechanisms leading to the observable outcome of a disturbed E/I balance combined with the computational limitations illustrates the limitations of any efforts aiming to map the complexity of psychiatric disorders with unspecific biomarkers. This example also points to the weakness of the assumptions underlying any categorization approach in psychiatry that is not based on specific biological measures. Staying with the example of E/I imbalance, a lack of biological specificity, makes it very difficult to assign the observed alterations to any specific pathophysiological process. Yet, such a distinction should most definitely play a role in the decision of what should be the primary treatment target. Two patients displaying similar psychiatric symptoms and E/I alterations would likely need different intervention strategies depending on if the E/I imbalance is causally related to their respective symptomatology or is mere reflection of other pathophysiological processes leading to the actual symptoms.

**Fig. 1:**
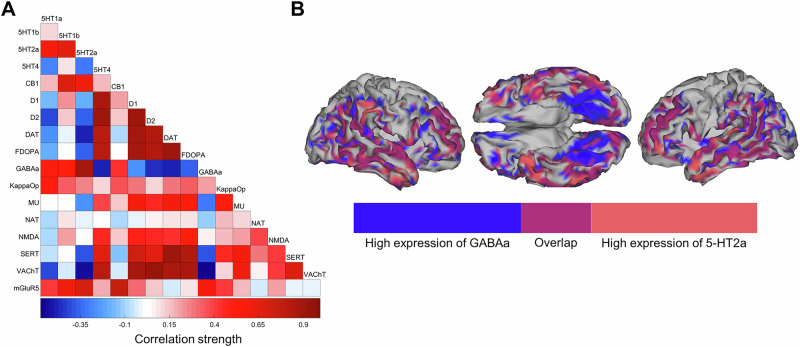
Spatial colocalization across major neurotransmitter systems. **A** Spatial correlation matrix of different neurotransmitter properties as derived from the positron emission tomography included in the JuSpace toolbox. **B** Exemplary visualization of the strong colocalization observed between GABAa and serotonergic 5-HT2a receptors. Regions with high expression of each receptor and their overlaps are displayed.

The E/I imbalance example also highlights the limitations of the dimensional approach. A neurotransmitter is never alone in a specific region or even brain circuit. In fact, most receptors and transporters across all major neurotransmitter systems display positive and often even very strong spatial co-localization (Fig. 1A). For example, the spatial distribution maps for GABAa and the serotonergic 5-HT2a receptor or the distributions of dopamine and serotonin transporters share about 80% of variance in each other’s whole-brain distribution [15] (Fig. 1B). This leads to a situation in which the disturbance of GABAa and 5-HT2a receptors in two patients might lead to virtually indistinguishable brain functional alterations, if measured using an unspecific imaging approach. In both the categorical and dimensional approaches, this similarity would erroneously indicate that both patients require the same type of treatment despite different pathophysiological mechanisms underlying their clinical symptoms. The issue becomes even more complicated when considering plasticity mechanisms, i.e. potential compensatory re-organization of other neurotransmitter systems in response to disease-inducing pathophysiological alterations [14]. Such secondary effects would appear to be temporally linked to the observed clinical symptoms without an actual causal relationship. That such neuroplasticity-based re-organization mechanisms exist and play a role is clearly demonstrated in stroke recovery studies where patients are often able to re-learn lost functions despite strong focalized impairments to specific brain regions [16].

The above examples illustrate how current categorical and dimensional approaches may severely underestimate the heterogeneity of the pathophysiological mechanisms underlying psychiatric conditions. Due to the continuous failure to identify reliable diagnostic biomarkers, recent studies moved towards the application of unsupervised clustering algorithms to identify biology-driven subtypes within or across psychiatric diagnoses [17–19]. However, these efforts do not address but simply move the problem to a different analysis level as they carry the same implicit assumptions that such distinct subtypes exist and that alterations observed in the same brain regions reflect the same pathophysiological mechanism. In the subsequent sections, we will demonstrate the problems with these assumptions from genetic, environmental and neuroimaging perspectives

## The genetic perspective

The strongest evidence against the above assumptions related to definition of distinct biological subtypes comes from genome-wide association studies. In the most recent of such studies, several hundred risk-loci have been reported to be separately associated with the increased risk of schizophrenia [20], major depression [21] and other psychiatric disorders [22, 23]. In addition, cross-diagnostic genome-wide studies reported substantial overlaps in risk loci associated with different psychiatric conditions [24]. The similarity of these genetic risks appears higher between psychiatric as compared to neurological disorders [25]. Whilst some of these risk loci have opposite directional effects being protective for some disorders and increasing risk for others, most show pleiotropic effects in their directional impact [26]. These disorder specific and pleiotropic risk loci clearly contribute to the heritability of psychiatric disorders. More importantly yet, they converge onto a variety of distinct molecular and cellular pathways [27].

The fact that such biological pathways are not mutually exclusive provides strong evidence for a many-to-many mapping problem in psychiatry. A person may carry risk alleles converging on any possible constellation of distinct biological pathways. As an example, a patient can simultaneously have genetic risks mapping to postsynaptic dopamine receptors, presynaptic serotonin reuptake and microglia function. Unless common mechanisms are identified that reduce the hundreds of known risk loci into a limited number of mutually exclusive constellations, any categorization or clustering effort of psychiatric diseases is likely subject to oversimplification. There is no biological reason to assume that a patient may not have pathophysiological alterations on more than one biological pathway leading to their clinical condition. Supporting this notion, different clustering efforts in various psychiatric populations and integrating different observation levels ranging from genetics over neuroimaging to clinical phenotypes resulted in highly heterogeneous findings ranging for example from 2 to 5 subtypes for autism [17, 28–31], 2 to 4 subtypes for schizophrenia [19, 32, 33] and 2 to 16 subtypes for major depression [18, 34, 35]. Whilst these differences may be partially attributable to deployment of different modalities for identification of the respective subtypes, they nonetheless illustrate the lack of convergence between genetics, imaging and clinical findings. Unless biological exclusiveness of the subtypes is clearly demonstrated the results of any such categorization or subtyping approaches are likely to remain futile.

Recognizing this problem, recent studies on polygenic risk scores (PRS) have started to move away from diagnosis-specific PRS towards parsing genetic risks based on their converging biological pathways. For example, several recent schizophrenia studies proposed single-ontology PRS that are specific to dopaminergic [36] and glutamatergic [37] neurotransmission as well as cell types including microglia, neurons and astroglia [38]. It remains to be shown if this proposed differentiation into different neurotransmitter systems or cell types as the units for the proposed biological pathways will be sufficient or if a more refined view, i.e. stratifying the single-ontology PRS into pre- and postsynaptic neurotransmission or different cell properties, is warranted. By establishing a closer and more specific link between genetics and observed imaging endophenotypes, such genetic risk parsing carries a lot of promise for dissecting the high heterogeneity observed in psychiatry [27]. More importantly, these genetic findings support a multidimensional view of the biology underlying the observed psychiatric symptoms. Staying with the schizophrenia example, a patient can carry an increased genetic risk on only one or all of the above single-ontology PRS being associated with their clinical symptoms.

## The environmental perspective

Environmental risk studies also support the notion of a multidimensional view of psychiatric disorders. For example, dozens of environmental risk factors are known for schizophrenia alone, starting from malnutrition and vitamin D deficiency in utero and infancy, to childhood trauma, smoking and substance abuse to social defeat and certain infections [39]. These risk factors act on entirely different time scales and through different biological mechanisms. They are also not specific to schizophrenia. Similarly long and often overlapping lists of environmental risk factors have been reported for most other psychiatric diseases [40, 41].

One way in which environmental risk factors operate is through epigenetic mechanisms, whereby the respective risks may interact with weakly acting genetic risk loci mapping to the different biological pathways. Through these interactions the environmental risk factors contribute to the individual risk of developing a specific psychiatric condition [42]. From a biological perspective, it is plausible to assume that most of the environmental risk factors converge in their mechanism of action to the same biological pathways as the ones defined by the single-ontology PRS. As for genetic risks, there is also no plausible reason to assume that such epigenetic interactions are mutually exclusive. A patient may be equally exposed to only one or all possible combinations of the known environmental risks. Each of these risks would map on its respective biological pathway thereby increasing the cumulative risk of developing specific clinical symptoms. Yet again, based on the above arguments, two patients who developed similar symptoms due to such distinct mechanisms are likely to require different treatments corresponding to their individual pathophysiology.

## Managing the many-to-many problem

It is fully understandable that clinicians desire a simple roadmap with, ideally, a single biomarker or algorithm for diagnosis and treatment selection. Yet, mounting evidence suggests that psychiatric diseases fall outside of such simplifications. The highly multidimensional nature of the known disease-related molecular and cellular pathways combined with the multidimensional nature of environmental risk factors leads to a continuum of possible combinations that can all lead to similar clinical phenotypes. Ultimately, this problem can and should be approached from multiple perspectives [43].

Parsing genetic risk scores into specific molecular and cellular pathways rather than diagnostic entities is certainly one of the starting points [27]. It is furthermore important to understand how a potential pathophysiology in each of these single-ontology pathways can contribute to the manifestation of specific clinical symptoms. Understanding that a specific person carries the increased risk for one, two or multiple of these pathophysiological pathways that can contribute to the observed clinical symptoms may substantially restrict the possible search space for that specific patient. Importantly, this restriction is not exclusive, but can only serve as a prior, because pathophysiology may also manifest in pathways without an individually increased genetic risk. At the same time, it is important to understand how each of the known environmental risk factors for the observed constellation of symptoms interacts with any of the single-ontology genetic pathways. For example, the increased risk of schizophrenia due to vitamin D deficiency has been linked to its action on the regulation of inflammatory and immunological processes [44]. Understanding such epigenetic effects would further restrict the possible search space for determining the individual pathophysiology and facilitate selection of possible interventions.

**Fig. 2:**
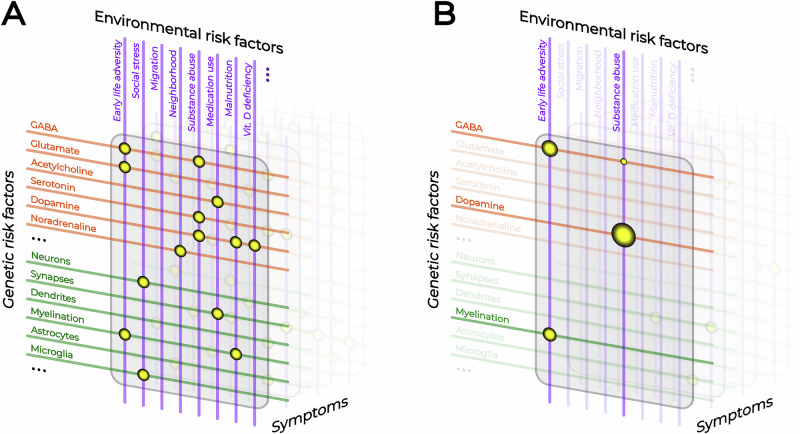
A schematic overview of the proposed vulnerability search grid approach. **A** A schematic representation of the general symptom-specific vulnerability search grid as defined by genetic and environmental risk factors. **B** A schematic representation of the individual vulnerability search grid as derived from individual genetic and environmental exposure.

The intersections of the biological pathways as determined by possible genetic and environmental risk factors can form a vulnerability search grid of all possible pathophysiological mechanisms underlying the manifestation of specific clinical symptoms (Fig. 2A). Restricting this search grid to the combinations of genetic and environmental risk factors to which an individual is or was actually exposed to can then provide a substantially reduced individual vulnerability grid for generating hypotheses about the actual mechanisms underlying their individual symptomatology (Fig. 2B). In this regard, not all environmental risk factors can be readily mapped onto a specific biological pathway. For example, certain complex risks, such as migration, are likely to exert multidimensional effects via a combination of biological and subjective psychosocial mechanisms. Two people with the same genetic risk and exposure to the same environmental risk factor may yield divergent outcomes depending on their subjective experience of that risk. An identical situation may be perceived as highly stressful by one individual, rendering them susceptible to stress-mediated modulation of dopaminergic transmission, whereas another individual may not perceive the same situation as stressful at all. Within this model, neither the common genetic nor the environmental risk factors are therefore deterministic in their mechanism of action. As discussed below, these vulnerability pathways can only narrow the search window for the specific pathophysiology, creating an opportunity for pathway-specific biomarkers to validate its presence based on probabilities derived from the vulnerability search grid.

## The potential for (neuroimaging) biomarkers in psychiatry

A major factor contributing to the, to date, limited usefulness of neuroimaging measures in psychiatry is, among other, the limited specificity of most of the proposed neuroimaging biomarkers. These limitations start with the classical brain mapping approaches testing for structural (e.g., cortical thickness) or functional [e.g., blood oxygen level dependent (BOLD)] alterations in specific brain-regions. Whilst somewhat useful for confirming the presence of a pathophysiological process, these measures are all extremely unspecific. Alterations in cortical thickness can be achieved through changes in myelination, actual neuropathology, hydration, starvation, physical exercise and many other known mechanisms. Similarly, alterations in BOLD activity or connectivity have been previously related to different tasks, states of mind, changes in the underlying neurotransmission, physical exercise, heart rate, breathing, neurostimulation and many other reported mechanisms. Any observed changes in such measures are therefore bound to be unspecific with respect to their interpretation. Any efforts of using these measures to directly derive single biomarkers, i.e. E/I imbalance or brain age, in the hope to reflect disease-specific pathophysiological processes are equally bound to become unspecific as various mechanisms can result in virtually indistinguishable perturbations of the respective metrics. To illustrate this point, E/I imbalance and accelerated brain age have been reported for every major psychiatric disease rendering them completely ineffective for differential diagnosis [13, 45].

Any initiatives aiming to change this status quo, therefore, need to identify biomarkers that are specific to the respective psychiatric conditions. Considering the above many-to-many mapping problem, such measures ideally also need to allow for individualized and pathology-specific interpretation of observed brain alterations.

**Fig. 3:**
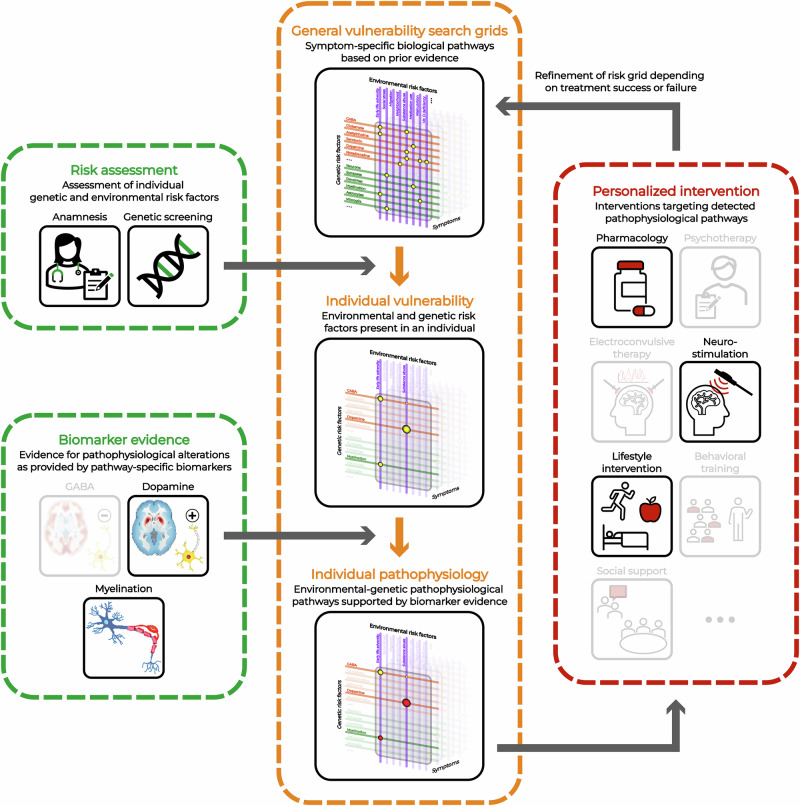
Overview of the proposed approach integrating symptom-specific genetic, environmental, and biomarker evidence. The general, literature-derived vulnerability search grid can be refined based on an individual’s genetic profile and environmental exposures, generating a personalized vulnerability risk grid. This risk grid can then be interrogated using pathway-specific biomarkers to detect actual pathophysiological alterations along these pathways. Based on this biomarker evidence, personalized interventions can be selected to target the identified pathophysiological alterations.

Ultimately, a holistic multidimensional approach that aims to measure pathology along the biological pathways, which contribute to the individual clinical symptomatology, should be the goal for biomarker development (Fig. 3). Ideally, such biomarkers should be specific to the biological pathways as derived from the above vulnerability grid of genetic and environmental factors. This can be achieved either through development of novel technologies allowing for an improved quantification of multimodal pathophysiology or through improvements in existing technologies by making them more specific to the relevant biological pathways.

Examples for the first approach are developments of novel target-specific tracers in nuclear medicine combined with the efforts of moving towards multi-tracer mapping approaches, i.e. through simultaneous administration of several positron emission tomography (PET) tracers or through combination of PET imaging with recently emerging deep-learning technologies to generate synthetic images of different biological properties [46]. Efforts to advance magnetic resonance spectroscopy towards measuring whole-brain multi-metabolomic profiles fall under this category, too [47]. All of these approaches strongly rely on overcoming substantial technical or other hurdles in technology development, i.e. dealing with increased radioactivity exposure in multi-tracer PET imaging or hardware limitations in case of whole-brain magnetic resonance spectroscopy.

At the same time, the second approach of adopting existing neuroimaging technologies to make them more sensitive to specific biological pathways appears more and more promising. Such efforts include recently proposed co-localization approaches testing for spatial associations between non-specific structural, functional or electrophysiological information with gene expression or nuclear medicine derived whole-brain atlases for specific biological pathways [15, 48]. Several research groups demonstrated that such approaches carry the potential for improving biological specificity of magnetic resonance imaging derived outcome measures [49–51]. Other efforts in this direction include the development of advanced biophysical compartment models and the adoption of multiparameter mapping acquisition protocols [52, 53].

Until more direct measures become available, adopting such optimized analysis approaches to readily available structural, functional and electrophysiological information may provide a starting point to move neuroimaging towards more specificity and more personalized evaluation of the underlying multidimensional pathophysiology. Importantly, these efforts can and should be complemented by careful validation of the underlying assumptions and integration of other biomarker modalities such as development of improved metabolomic panels, pluripotent stem cells or brain organoids to gain potentially more specific causal insights into the individual pathophysiology.

## Integration of biomarkers, genetics and environment

**Fig. 4:**
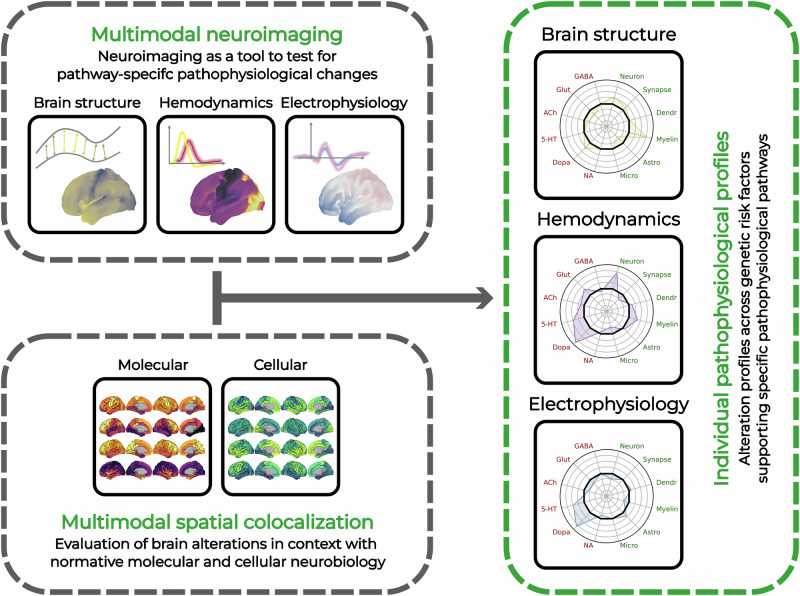
Schematic representation of the spatial co-localization approach for deriving biological pathway-specific information from multimodal neuroimaging.

Obtaining neuroimaging or other biomarker fingerprints that are specific to the underlying biological pathways carries several advantages with respect to integration with the above described vulnerability search grid defined by the combination of genetic and environmental risk factors (Fig. 4). First of all, such biomarkers would provide strong subject-specific evidence for the actual pathophysiological manifestation in the respective gene-by-environment biological vulnerability pathway. Moreover, in the context of the strong positive colocalization of many of the molecular systems and considering the presence of potential compensation mechanisms, even a highly specific biomarker cannot differentiate between the actual causal and adaptational effects in the brain. In this regard, combining the evidence from biomarkers with the vulnerability search grid defined by genes and environment may substantially facilitate such a discrimination. It is reasonable to assume that, for an individual patient, alterations in a specific biological pathway are more likely to be causally related to their disease manifestation when meeting specific criteria. These include being supported by the patient’s genetic risk, being linked to known environmental exposures, and having established associations with the observed clinical manifestation. In contrast, biomarker alterations that lack such supportive evidence may be less likely to have contributed to the patient’s condition. Such a step-wise procedure would facilitate identification of the actual causal pathophysiological mechanisms underlying each patient’s individual clinical condition, reducing the many-to-many problem to a clinically manageable approach.

An important aspect that is often disregarded in biomarker research in psychiatry is the differentiation between state and trait pathophysiology. States are expected to reflect disease dynamics, i.e. the magnitude of clinical symptoms and would be expected to improve following successful intervention. In contrast, traits would typically precede disease onset providing evidence of an increased risk for a specific clinical condition. In the above vulnerability search grid, this distinction is important for selection of appropriate interventions. It is plausible to assume that even with a strong causal relationship, trait-like pathophysiology that evolves over decades is unlikely to be easily modifiable on the time scale of typical interventions. Long-term interventions or aiming for compensation may be the only viable treatment options here. On the contrary, one may expect quick recovery when normalizing pathophysiological brain states. Although this distinction may not always apply, it provides a useful framework for aligning intervention strategies with the temporal characteristics of the underlying pathology. An integrative view on biomarkers, genetics and environment is necessary to advance understanding of these temporal associations.

## Integration with current diagnostic frameworks

Importantly, an integrative framework that combines evidence from genetics, environment, and biomarkers does not contradict the routine diagnostic practices of modern psychiatry. At its core, this approach is agnostic to existing diagnostic categories, instead emphasizing the individual, potentially unique, multimodal pathophysiology underlying observed symptom constellations. Given the likely limited number of biological pathways contributing to specific symptoms, this framework anticipates convergence of certain constellations across patients, thereby enabling more precise definitions of psychiatric disorders grounded in biology. Crucially, however, it also recognizes that most patients may fall within a multidimensional spectrum characterized by distinct, coexisting biological pathways. Traditional taxonomies can thus remain valuable for initial assessment and everyday clinical care, while treatment strategies must shift from a one-size-fits-all model to personalized, biology-informed, multidimensional interventions targeting these patient-specific mechanisms. In this way, the proposed integrative approach not only refines current interventions but also facilitates the discovery of novel therapies, as illustrated below.

## Advancing clinical interventions

Despite an increased unmet medical need, drug development in psychiatry has experienced major setbacks in the past with many major pharmaceutical companies having withdrawn from such efforts over the past decades due to limited success in developing new interventions [54]. Particularly the use of diagnostic constructs that are ill-suited or unrelated to the underlying biological mechanisms has been highlighted as a major contributing factor to the frequent failures of novel interventions in clinical trials [55]. Considering the many-to-many problem illustrated above, any inclusion criteria for such clinical trials that are based on the diagnosis or even specific symptom dimensions are bound to result in inclusion of patients with distinct constellations of biological pathways contributing to their respective clinical symptoms. In such a scenario, the effect of any drug with a pre-specified mechanism of action would be substantially diluted as the drug would be only effective in a subpopulation of patients with a matching pathophysiology. Indeed, such dilution effects have been suggested as a major explanatory mechanism for low effect sizes observed in treatment trials of depression [56].

Adopting a many-to-many perspective on individual pathophysiology in psychiatric disorders may open novel avenues towards improved applications of existing and development of novel, more effective interventions. First of all, it is important to understand if and how existing interventions act or interact with each specific biological pathway. Whilst such relationships are relatively straightforward for most pharmacological interventions, other treatment options, such as electroconvulsive therapy, neurostimulation, psychotherapy or environmental interventions, would need to be carefully evaluated with respect to their underlying biological mechanisms of action. Having established such a mapping of interventions to biological pathways carries several advantages. Instead of a trial and error approach moving from first line to second or third line of treatment, interventions could be tailored to individual pathophysiology through a targeted combination of different interventions aiming to restore or compensate for the individual multidimensional pathophysiology along the affected biological pathways. Such an approach may initially appear restrictive for the possibility of conducting large-scale clinical trials. However, the shift of focus does not reduce the pool of available patients, but rather opens the window for cross-diagnostic interventions with potentially larger effect sizes due to a better match up of interventions with the underlying pathophysiology. Importantly, the successes and failures of such intervention trials could help refine the vulnerability search grid by providing evidence for or against a causal relationship between specific interventions and biological pathways.
